# The Effect of Intracoronary Infusion of Autologous Bone Marrow-Derived Lineage-Negative Stem/Progenitor Cells on Remodeling of Post-Infarcted Heart in Patient with Acute Myocardial Infarction

**DOI:** 10.7150/ijms.42561

**Published:** 2020-04-06

**Authors:** Małgorzata Peregud-Pogorzelska, Krzysztof Przybycień, Bartłomiej Baumert, Maciej Kotowski, Ewa Pius-Sadowska, Krzysztof Safranow, Jarosław Peregud-Pogorzelski, Zdzisława Kornacewicz-Jach, Edyta Paczkowska, Bogusław Machaliński

**Affiliations:** 1 Department of Cardiology, Pomeranian Medical University, Powstańców Wlkp. 72, 70-111 Szczecin, Poland; 2 Department of General Pathology, Pomeranian Medical University, Powstańców Wlkp. 72, 70-111 Szczecin, Poland; 3 Department of Biochemistry and Medical Chemistry, Pomeranian Medical University, Powstańców Wlkp. 72, 70-111 Szczecin, Poland; 4 Department of Paediatric Oncology, Pomeranian Medical University, Unii Lub. 1, 71-252 Szczecin, Poland

**Keywords:** infarct, left ventricular failure, lineage-negative cells, remodeling, stem/progenitor cells

## Abstract

**Introduction**: Regenerative capacity of the heart is limited, and the post-infarct left ventricle (LV) dysfunction is associated with poor prognosis. Administration of stem/progenitor cells (SPCs) is a promising approach for cardiac regeneration.

**Objectives**: In the study, we assessed LV function and post-infarcted remodeling in patients with ST-elevated myocardial infarct (STEMI) who received autologous lineage-negative (LIN^-^) SPCs.

**Patients and methods**: Patients with STEMI and one-vessel coronary artery disease treated with percutaneous revascularisation were divided into study group (LIN^-^ group, 15 patients) that received standard therapy and autologous BM-derived LIN^-^ SPCs and control group (standard therapy group, 19 patients). The cells were administered intracoronary 24 hours after STEMI. The follow-up was 12 months with subsequent non-invasive tests and laboratory parameter evaluation on days 1^st^, 3^rd^, and 7^th^ as well as at 1^st^, 3^rd^, 6^th^ and 12^th^ month after STEMI.

**Results**: All procedures related to SPCs administration were well tolerated by the patients. In 12-month follow-up, there were no major adverse cardiac events connected with LIN^-^ SPCs administration. During 12-month follow-up, 9 patients from LIN^-^ group (Responders) achieved an improvement in LV ejection fraction (>10% after 12 months) with no signs of unfavorable LV remodeling. Laboratory parameters analysis showed that Troponin T levels were significantly lower until day 7^th^ in the Responders group, while brain natriuretic peptide (BNP) level remained significantly lower from day 3^rd^ to 12^th^ month respectively.

**Conclusions**: Intracoronary infusion of autologous BM-derived LIN^-^ stem/progenitor cells is feasible and safe for patient. Improvement in LV function and prevention of unfavorable remodeling in the 60% of study group seems relatively promising. Stem cell-based therapy for cardiac regeneration still needs more accurate and extensive investigations to estimate and improve their efficacy.

## Introduction

Structural and functional changes occurring in the myocardium as a result of myocardial infarct have long been a subject of interest for many researches. The cardiac muscle remodeling is a dynamic process, often taking many years and it may lead to development of heart failure which at present is the most important challenge for cardiologists. It can be defined as a group of complex morphological changes on different levels of heart structure, cell, tissue, and organ 1. This process is initiated with changes in specific gene expression in response to prolonged pathological strain in myocardium as well as a structural regulation, which together with homeotropic and heterometric regulations is an adaptive mechanism allowing for heart functioning under changed conditions 1. Prompt reperfusion of the infarct-related artery (IRA) is of great importance to rescue affected myocardium and limit the infarct size. However, despite restoring IRA patency, unfavorable remodeling of the LV and heart failure development can be observed in as many as 30% of patients during early and long-term follow-up 2, 3. Damage of cardiomyocytes resulted in a release of specific cardiac markers of myocardium injury into blood stream. Sequential measurements of cardiac troponins provide also very useful information about infarct size in myocardial infarction patients 4. It has been demonstrated that creatine kinase (CK-MB) measurement is useful in estimating infarct size and left ventricular ejection fraction (LVEF) 5. Previous studies suggest that post-infarct dilatation of the left ventricle occurs more frequently in patients that underwent fibrinolytic treatment (30% of patients) compared to those treated with percutaneous revascularization method (21%) 2,6.

Regenerative capacity of the heart is limited, and the post-infarct LV dilatation is associated with LV dysfunction and poor prognosis. There are various endeavors to improve post-infarct LV function by promoting the replacement of the lost cardiomyocytes or by activating cardiac repair. For example, intracoronary delivery of autologous stem/progenitor cells (SPCs) is a promising adjuvant therapeutic strategy to improve LV function for patients with acute myocardial infarction (AMI) 7, 8. However, published data from clinical trials conducted so far showed inconsistent results in regard to the effectiveness of stem cell-based therapy 9-14. Our understanding of pathomechanisms of stem cell-based therapy is still limited. At the beginning, a direct replacement of damaged cardiomyocytes by transplanted stem/progenitor cell transdifferentiation, differentiation or cell fusion process has been postulated 14, 15. Next, secretion of growth factors has been considered to contribute to the functional benefit of treatment using SPCs 16-19. Additionally, it has been also demonstrated that cell to cell contact was pivotal to the functional benefits of cell therapies 20.

In the study, we assessed the safety and efficacy of intracoronary administration of autologous BM-derived LIN^-^ cells enriched in SPCs 24 hours after percutaneous coronary intervention (PCI) in the patients with STEMI. The study goal was to investigate long-term effects (up to 1 year) of single LIN^-^ cells administration on heart function in STEMI patients.

## Patients and methods

### Patients

Our study was a prospective, open-label, nonrandomized clinical trial. Patients were recruited from the Department of Cardiology at Pomeranian Medical University in Szczecin. The study was approved by the Ethics Committee of the Pomeranian Medical University in Szczecin (Poland, BN-001/122/05, 20.06.2005) and performed in accordance with the Declaration of Helsinki. All patients provided written informed consent.

Between December 2010 and June 2014, 34 patients were allocated to 2 groups: 15 patients in a study group beside standard therapy were treated with autologous LIN^-^ cells; 19 in a control group were given standard treatment solely. Primary angioplasty was performed according to the practice guidelines in patients with fully obstructed coronary artery. Only the infarct-related artery was supplied.

### Inclusion criteria

In the study, we settled the following criteria for enrolled patients:

a) age less than 65 years,

b) occurrence of a typical angina pectoris lasting at least 30 minutes and the appearance of chest pain up to 12 hours before admission to the clinic,

c) elevation of the ST segment at point J > 0.2 mV in at least two adjacent leads from V1 to V3 or > 0.1 mV in other electrocardiogram (ECG) leads,

d) first ever myocardial infarction,

e) ejection fraction (EF) ≤ 45% in day 0 echocardiographic examination,

f) single-vessel coronary disease in coronary angiography qualified for coronary angioplasty with stent implantation.

### Clinical assessment

On admission, the clinical condition of all enrolled patients was analyzed according to Killip Kimball's classification and ultrasonography. Subsequently, coronary angiography and percutaneous revascularization with the implantation of drug eluting stent (DES) to infarcted artery were carried out with TIMI grade 3 flow. Coronarography was performed using the Judkins technique on the Integris HM hardware (Philips Allura Xper FD 10 System) and analyzed using DICOM 3 software. The initial and post-angioplasty coronary flow was assessed in keeping with the TIMI.

### Preparation of autologous BM-derived LIN^-^ cells

BM aspirates were obtained from 15 patients in study group under local anesthesia from the posterior iliac crest within 24 hours after PCI. Mononuclear cells (MNCs) were obtained from harvested BM using centrifugation over Gradisol L (Polfa, Kutno, Poland) as described elsewhere 21. Subsequently, the cells were incubated with anti-human antibodies conjugated with microbeads using a Lineage Depletion Kit (Myltenyi Biotec, Auburn, AL, USA) and isolated according to manufacturer's protocol as described 17, according to the good medical practice conditions. All isolated LIN^-^ cells were suspended in 2 ml sterile phosphate buffered saline and transported to the catheterization laboratory. The purity of the enriched lineage-negative cells was evaluated by flow cytometry according to the manufacturer's protocol (Myltenyi Biotec, Auburn, AL, USA). Briefly, aliquots of the cell fractions were stained with a fluorochrome-conjugated antibody against CD133 and against CD34, for the staining of hematopoietic progenitor cells. Each time lineage-negative cell viability was evaluated by the 0.5% trypan blue exclusion assay and exceeded 95%. Table 1 shows the phenotypic characterization of administered LIN^-^ cells. In our previous work, by employing flow cytometry, we have performed a phenotypic characterization of the population using flow cytometry, showing that LIN^-^ cells contain about 12% of CD34^+^ cells and CD133^+^ cells, 2% of CD34^+^CD133^+^CD144^+^ cells (endothelial progenitor cells) and very small percentage (0.01%) of CD105^+^CD73^+^CD90^+^CD45^-^CD34^-^CD11b^-^CD19^-^HLA^-^DR^-^ cells (mesenchymal stem cells) 17.

### LIN^-^ Cell Administration

The injection of isolated BM-derived LIN^-^ cells was performed up to 24 hours after PCI. LIN^-^ cells were gently transferred into the infusion syringe and infused via the central lumen of an Over-The-Wire balloon dilatation catheter (Boston Scientific Emerge TM) into the infarct-related coronary artery. After placing the balloon of the OTW catheter within the previously implanted stent, the balloon was inflated inside the stent at a low pressure to transiently interrupt antegrade blood flow during the infusions. The prepared suspension of BM-derived LIN^-^ cells was injected through the catheter flow channel distal to the balloon. Deflation of the balloon and re-perfusion was performed 2 minutes after the infusion. After cell administration, close observation identified clinical changes and/or possible complications. The control group was not subjected to another coronarography within 24 hours due to ethical reasons.

### Follow-up visits and assessment

Subsequent assessment during hospitalization was carried out on days 1^st^, 3^rd^, and 7^th^. The study visits were scheduled at 1^st^, 3^rd^, 6^th^ and 12^th^ month after STEMI for the clinical and functional evaluation. Major adverse cardiac events (MACE) were assessed. The MACE were defined as the composites of death, repeat myocardial infarction, stent thrombosis, major arrhythmia, repeat target vessel revascularization. In order to assess life-threatening arrhythmia patients included in the study in the first day and after 6 and 12 months underwent a 24-hour Holter ECG. Oxford apparatus and software were used for the study (Oxford Pol Sp. z o.o., Poland).

### Assessment of left ventricular function

Echocardiography was conducted to measure end-diastolic volume (LVEDV), end-systolic volume (LVESV) on day 1^st^, 3^rd^, 7^th^, as well as at 1^st^, 3^rd^, 6^th^, and 12^th^ months after baseline observation. Echocardiography was performed each time by the same professional blinded to the treatment arm using the Acouson 128 XP/10c apparatus with cardiac transducer 2/5/3,5 MHz. The heart function parameters data were estimated with the use of standard projections: apical four-chamber, dual-chamber long, short axis in two-dimensional mode, and color M-mode Doppler. The results were recorded on a CD and analyzed on- and offline. Left ventricular function was assessed based on left ventricular ejection fraction (LVEF), end-diastolic volume (LVEDV), end-systolic volume (LVESV) obtained with Simpson's two-dimensional method in four-chamber and two-chamber apical projection using Acouson software.

### Statistics

Chi-square or Fisher's exact test was used to compare qualitative variables, and Mann-Whitney test was used to compare quantitative variables between groups. Since the number of patients in each group was too low to assess reliably normality of distributions of quantitative variables, non-parametric tests were used, and data are presented as median (interquartile range - IQR). Differences of parameters measured on day 0 and on subsequent days of observation in each patient (delta values) were calculated and compared between groups to study the dynamics of changes. Significance of the differences within each group was assessed with repeated-measures Friedman ANOVA which was followed, in the case of significant (p < 0.05) differences between time points, by Wilcoxon signed-rank test for comparison to baseline (day 0) values. Spearman's rank correlation coefficient (Rs) was used to measure strength of associations between quantitative variables within groups. p < 0.05 was considered statistically significant.

## Results

### Baseline characteristics

The study included male patients with STEMI with one-vessel coronary artery disease and percutaneous revascularisation. Study design was not randomized. However, the clinical characteristics of recruited patients from LIN^-^ group and standard therapy group were similar (Table 2). One of the study objectives was evaluation of long-term effects (up to 1 year) of single LIN^-^ cells administration on heart function in STEMI patients. Therefore, patients from study group who achieved an improvement in EF >10% after 12 months compared to day 0 were assigned to the Responders group (n = 9), while the others were assigned to the Non-responders group (n = 6). Characteristics of Responders and Non-responders is presented in Table 3.

### Clinical characteristic of STEMI

All recruited patients were patients with one-vessel coronary disease. Time intervals from chest pain onset to treatment and restoration of coronary blood flow was 6.2 (average) hours for both groups. In LIN^-^ group, 10 patients had left anterior descending artery (LAD), 2 patients had left circumflex artery (LCX) and 3 patients had right coronary artery (RCA) occluded. In the standard therapy group, 14 patients had LAD, 1 patient had LCX and 4 patients had RCA occluded. Primary PCI was carried out in all patients. During the procedure the infarct-related artery was opened and TIMI 3 flow was observed. There were no significant differences in procedural characteristics. Pharmacological treatment was initiated according to current guidelines shortly after primary percutaneous coronary intervention (PCI).

### Baseline ultrasonography

To assess the effect of intracoronary LIN^-^ SPCs application on LV function echocardiography with measurement of essential cardiac parameters was performed. The echocardiography was conducted on day 1^st^, 3^rd^, 7^th^, as well as at 1^st^, 3^rd^, 6^th^, and 12^th^ month after baseline observation. On day 0 there were some differences between study and control group of patients. Patients in the control group had a lower EF (median 35 (3.75) % vs 40 (5.75) %, p = 0.047), higher LVEDV (median 151.5 (31.75) ml vs 128.5 (39.25) ml, p = 0.025). We observed also higher LVESV (median 90.5 (16.0) ml vs 74 (22.25) ml, p = 0.043) and higher left ventricular internal dimension at end-diastole (LVIDD) (median 56 (4.0) mm vs 51 (4.0) mm, p = 0.007) at the day 0. Comparing Responders to Non-responders from LIN^-^ group, there were no significant differences in baseline EF, LVEDV and LVIDD. Only LVESV was significantly lower in Responders group (median 72.5 (16.3) ml vs 76.0 (35.5) ml, p = 0.011) (Table 4).

### Baseline laboratory parameters

Laboratory tests were performed on the obtained peripheral blood samples in different time points. There were no differences between study and control group in baseline CK-MB, Troponin T, brain natriuretic peptide (BNP) levels and c-reactive protein concentration. In the LIN- group, Responders had significantly lower initial levels of Troponin T, CK-MB and BNP (Table 5).

### Cell-based intervention

In LIN^-^ group a mean (SD) of 8.37 (7.8) ×10^6^ autologous BM-derived LIN^-^ SPCs have been infused in the infarct-related artery within 24 hours after PCI.

### Changes in cardiac parameters during 12-month follow-up

Echocardiography follow-up was performed on days 1^st^, 3^rd^, 7^th^, and 1^st^, 3^rd^, 6^th^ and 12^th^ months after myocardial infarction. The comparison of EF, LVEDV, LVESV between study and control group in subsequent time points did not differ significantly. However, in the Responders group significantly higher EF was observed from day 3^rd^ to 6^th^ month. Parallel, LVEDV and LVESV were significantly lower from month 3^rd^ to 12^th^ in Responders group. The exact measurements of selected parameters are shown in Table 4.

Interestingly, at 6^th^ and 12^th^ month we observed an unfavorable remodeling of LV in standard therapy group compared to LIN^-^ group. We noticed an increase in its diameter (median (IQR); 6-month — 55.5 (10.3) mm vs 49.5 (9.8) mm, p = 0.05, 12-month — 58 (9.0) mm vs 53 (9.5) mm, p = 0.05) in standard therapy group compared to LIN^-^ group. A similar relationship was observed in favor of Responders group from day 3^rd^ to 12^th^ month (Table 4).

To summarize, we did not observe differences in main LV function parameters between LIN^-^ group and standard therapy group. Concurrently, we did observe features of unfavorable remodeling of LV in standard therapy group in comparison to patients who received LIN^-^ SPCs.

### Holter 24-hour ECG

In order to assess life-threatening arrhythmia patients included in the study in the first day and after 6 and 12 months underwent a 24-hour Holter ECG. In LIN^-^ group, supraventricular arrhythmia occurred in the form of single ectopic beats, bigeminy occurred in one person at 6 months after AMI. No couplets were observed. In contrast, episodes of atrial fibrillation occurred in one patient at 6^th^ month after AMI. Ventricular arrhythmia occurred in the form of single ventricular ectopic systoles on the 1^st^ day of AMI, averagely 152 beats per day. There were on average 170 ectopic beats per day at 6^th^ month and 115 per day at 12^th^ month. There were also several couples and bigeminy in one patient at 6^th^ month. Ventricular tachycardia has not been observed. In the control standard therapy group, single ventricular ectopic beats were also observed, on average 93 per day on 1^st^ day, 55 at 6^th^ month, and 215 per day at 12^th^ month. There were no arrhythmias that would be an indication for implantable cardioverter-defibrillator implantation in patients from LIN^-^ and standard therapy group.

### Laboratory measurements during 12-month follow-up

**Figure 1:**
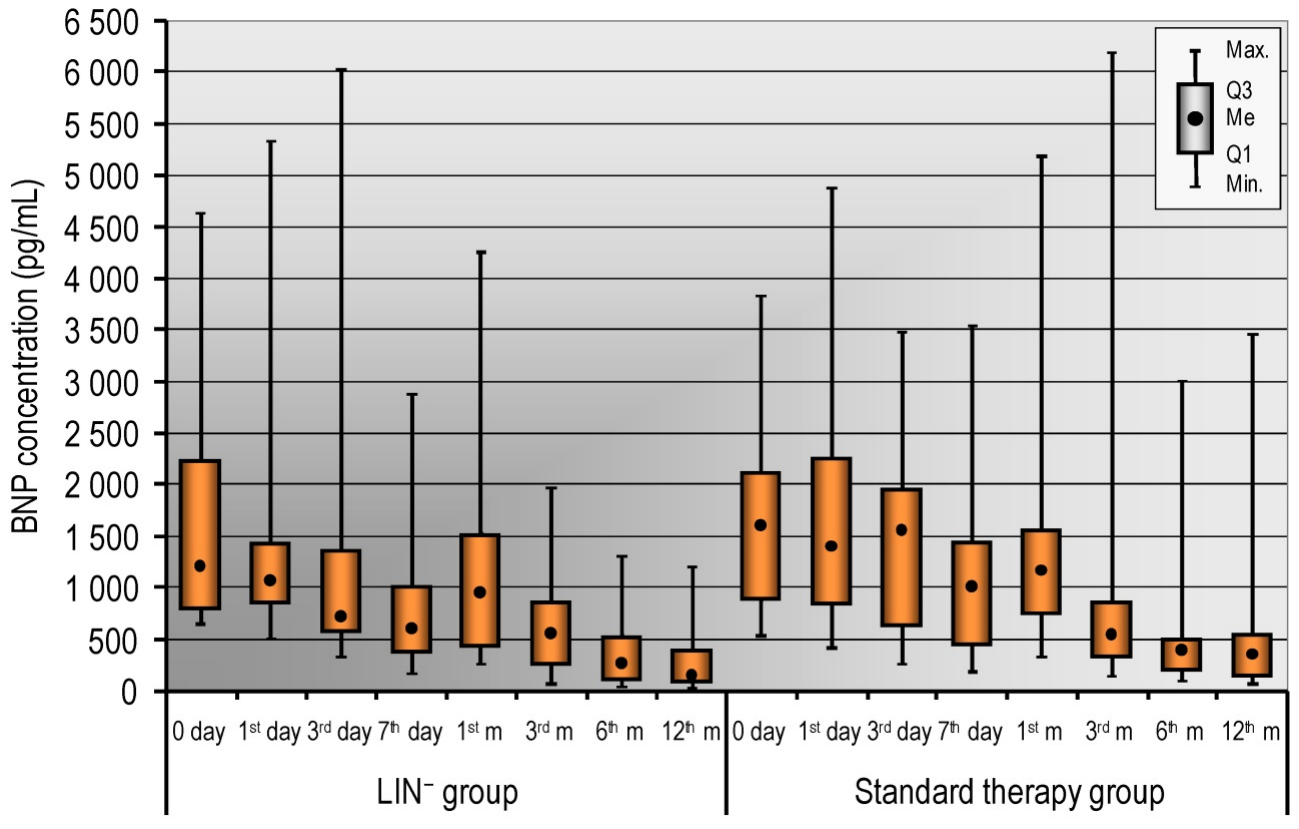
The serum levels of BNP in LIN^-^ group and in the standard therapy group (0, 1^st^, 3^rd^, 7^th^ day and in the 1^st^, 3^rd^, 6^th^, 12^th^ month). Data are presented as median (lower - upper quartile).

Infarct size is the strongest determinant of post-infarction LV function, the compensatory mechanisms and in the long-term adverse volumetric changes that occur in response to a depressed ejection fraction 4. However, the infarct size estimation by cardiac troponins is useful also when integrated with other markers of risk. In the study, we did not notice any differences in cardiac enzyme levels including CK-MB, troponin levels between LIN^-^ and standard therapy group. Well documented in relevant literature is the prognostic value of natriuretic peptides. Thus, we measured the level of BNP and we have presented the results in Figure 1. We did not observe statistically significant differences in the level of BNP in LIN^-^ and standard therapy group. However, we noticed that a decrease of BNP concentration has tendency to be more rapid in patients who received LIN^-^ stem/progenitor cells compared to standard therapy group (Figure 1). Detailed analysis of the LIN^-^ group showed that Troponin T levels were significantly lower until day 7^th^ in the Responders group. While, BNP level remained significantly lower from day 3^th^ to 12^th^ month in the Responders group (Table 5).

### Safety and clinical outcomes

During procedure and 12-month follow-up we performed strict observation of patients in regard to the occurrence of MACE. All procedures related to the BM aspiration and LIN^-^ cell administration were well tolerated by patients. We did not observe any inflammatory reactions or bleeding complications at the site of iliac puncture after BM aspiration. There was no angina during balloon inflation performed during LIN^-^ cell infusion. Patients did not experience serious procedural complications associated by infarct-related artery administration of cells, such as ventricular arrhythmias, thrombus formation or artery dissection, periprocedural myocardial infarction. In 12-month follow-up, there were no MACE such as death, recurrent myocardial infarction, stent thrombosis, major arrhythmia, repeated target vessel revascularization in LIN^-^ group. 24-hour ambulatory ECG (Holter) monitoring did not show significant arrhythmic events.

To sum up, there were not significant differences in clinical outcomes in patients who received LIN^-^ cells compared with patients receiving standard therapy.

## Discussion

AMI remains the most common cause of heart failure that is a determinant of adverse prognosis in the STEMI patients 22. PCI has improved early survival after STEMI. However, its impact on the incidence of following heart failure is debated 22. On the basis of available literature, it is estimated that despite successful PCI and restoring flow in IRA, unfavorable remodeling of the LV and heart failure development can be noticed in 30% of patients during early and long-term follow-up 2, 3. LV remodeling is characterized by progressive dilatation and distortion of ventriculi geometry connected with the deterioration of global contractile function. The important factors associated with post-infarct remodeling are the increase in the wall stress that induces cardiac hypertrophy of non-infarcted regions and activation of matrix metalloproteinases that resulted in degradation of collagen and increases LV dilatation. For the definition of remodeling, the criterion of the individual increase of 20% of LV end-diastolic volume between the acute phase and the 6-month follow-up is used 2. Patients after STEMI with LV remodeling who met the criterion had worse long-term outcome in the study of Bolognese *et al*. It is well known that levels of BNP and NT-proBNP increase rapidly within the first 24 hours and then decrease gradually in the course of AMI 23. In the relevant literature, a strong correlation between BNP or NT-proBNP levels and EF has been emphasized 24. In the study, we did not observe statistically significant differences in the level of BNP in LIN^-^ and standard therapy group. However, due to the lack of group randomization, these results should be interpreted with caution. Interestingly, we noticed a tendency to more rapid normalization of BNP level during the first days in patients receiving LIN^-^ stem/progenitor cells compared to standard therapy group. Responders group separated from the LIN^-^ group, with the improvement of EF > 10% after 12 months, had significantly lower initial levels of Troponin T, CK-MB and BNP. Detailed analysis during 12-month follow-up showed that Troponin T levels were significantly lower until day 7^th^ in the Responders group, while BNP level remained significantly lower from day 3^rd^ to 12^th^ month respectively.

Hence, many efforts to prevent LV remodeling and promote myocardial repair have been undertaken in clinical trials. Development of stem cell biology research that we could observe over the past decades resulted in several types of SPCs available for investigation in regenerative medicine. In acute phase of STEMI, an increase in circulating SPCs has been reported in a number of studies 25. These cells are released from BM and attracted into site of damage through gradient of chemoattractants such as vascular endothelial growth factor (VEGF), stromal derived factor-1 (SDF-1) 26. Administration and homing of SPCs might have synergistic effect in process of cardiac repair. It may play a role in a succeeding post-infarct remodeling of myocardium.

A number of therapeutic approaches have been tested in trials concerning cardiac repair and remodeling following cardiomyocyte damage. Different cell sources and cell types such as bone marrow cells, stem and progenitor cells, mesenchymal as well as resident cardiac stem cells have been employed in many ongoing and completed clinical trials. In our study, we used autologous BM-derived LIN^-^ stem/progenitor-enriched population of cells. Autologous cells are not immunologically rejected. The BM cells are easy to obtain and the isolation procedures of the stem/progenitor cells from BM are well-established. BM stem cells can be divided into two main subtypes of cells, depending on their function and surface markers: bone marrow hematopoietic stem cells (BM HSCs) characterized by the expression of CD34, CD45, and CD133, and mesenchymal bone marrow stem cells (MSCs), which express CD73, CD90, and CD105 27. In our study, we employed for the first time unique population of lineage-negative cells. We used immunomagnetic negative selection to deplete BM mononuclear cells (MNCs) of hematopoietic lineage marker-expressing mature cells. LIN^-^ cells are heterogeneous population which is highly enriched in stem cells and progenitor cells. We have also previously demonstrated that human umbilical cord blood-derived LIN^-^ cells strongly and specifically express trophic factors such as neuroprotective and angiogenic factors. Next, we have shown that autologous BM LIN^-^ cells administered intrathecally into cerebrospinal fluid could be used as feasible and safe adjuvant therapy for patients with amyotrophic lateral sclerosis 28. Beneficial effects of SPCs might be explained by paracrine and trophic effects of growth and chemotactic factors, cytokines that are released by the cells. According to adjuvant therapy SPCs might be a rich source of humoral factors such as cytokines and growth factors that regulate cardiac cell function. It has been also hypothesized that administered cells provide an immunomodulatory effect involving among others the macrophage polarization switch from M1 to M2 macrophages which further perform anti-inflammatory effects. It is suggested to be a key event in myocardium repair.

Considering technical aspects of the procedure, different routes of cell administration have been investigated in clinical trials and each of them has limitations. Intramyocardial injection allows the administration of high number of cells and seems to result in good cell retention within myocardium but it is very invasive technic. Other technics feasible to cell administrations include e.g. catheter-based intramyocardial administration, trans-endocardial injection, trans-coronary venous injection, intravenous infusion. In our study, we administrated LIN^-^ SPCs into the coronary artery. An advantage of intracoronary artery administration is a homogeneous distribution of cells inside large myocardial regions. The procedure is also less complicated than intramyocardial injection. Limitations are connected with probable low retention of infused cells 29. In our hands, the procedure of intracoronary delivery of LIN^-^ SPCs was feasible and safe for patients. We did not observe substantial complications during and immediately after procedure.

There were different endpoints for evaluation in various clinical studies, such as LV ejection fraction, maximum oxygen consumption, brain natriuretic peptide or myocardial perfusion 30. In our 12-month follow-up, we assessed cardiac enzyme levels including CK-MB, troponin levels, NT pro-BNP, ultrasonography parameters, Holter 24-ECG on days 1^st^, 3^th^, 7^th^ and months 1^st^, 3^rd^, 6^th^ and 12^th^.

So far, a number of trials have been performed with use of SPCs in patient with myocardial infarction. Unfortunately, published data has demonstrated inconsistent results in regard to effectiveness of these experimental therapeutic options. In the study, we did not observe an improvement in ejection fraction of LV in patients receiving LIN^-^ cells compared with control group. However, we did not notice the signs of unfavorable LV remodeling in LIN^-^ group whilst the unfavorable changes such as the increase in LV diameter after 6 and 12 months in the control group. Taking Responders group into consideration, significantly higher EF was observed from day 3^rd^ to 6^th^ month, while LVEDV and LVESV were significantly lower from month 3^rd^ to 12^th^, respectively. A similar prevention of remodeling was observed in favor of Responders group from day 3^rd^ to 12^th^ month. We employed for the first time BM-derived LIN^-^ SPCs in patients with STEMI. There is lack of published data concerning the use of this type of BM-derived cells. In other published studies, non-separated BM cells have been administered. For example, in the relatively small study TOPCARE-AMI individuals were randomized to receive either circulating blood-derived or BM-derived progenitor cells directly into the coronary artery after AMI. A significant improvement in LV function and markedly lower ventricular volumes were found in comparison to a nonrandomized matched control group 31. In another study, BOOST-trial Wollert *et al*. demonstrated that intracoronary autologous BM cell infusion after AMI markedly improved LVEF after a 6-month follow-up 32. In the study REPAIR-AMI, a significant improvement in myocardial performance (LVEF obtained by “eye-balling” method) has been demonstrated after administration of BM-derived progenitor cells. After one year, a significant reduction in clinical end points in the study group (death, relapse of myocardial infarction, renewed revascularization procedure) has been shown 33. In our study, in echocardiography follow-up, we did not observe either the improvement in main LV function nor features of unfavorable remodeling of LV parameters in LIN^-^ group compared to standard therapy group. Additionally, there were no MACE connected with LIN^-^ SPCs administration. Comparing Responders to Non-responders from LIN^-^ group, there were no significant differences in baseline EF, LVEDV and LVIDD. However, in the Responders group significantly higher EF was observed from day 3^rd^ to 6^th^ month, while LVEDV and LVESV were significantly lower from month 3^rd^ to 12^th^ respectively.

Exact mechanisms of cardiac repair by transplanted cells are still discussed. It is hypothesized that administrated cells can differentiate into cardiomyogenic/vasculogenic direction. Moreover, these cells can indirectly stimulate the regenerative processes through paracrine, immunomodulatory effects by supply/secretion of soluble cytokines and growth factors 34, 35. Paracrine signaling seems to be responsible for at least part of the therapeutic effects observed in stem cell-based studies. A number of observations is supporting this concept of the secretory ability of SPCs. They can secrete several cytokines such as TNF-alpha, IL-6, IL-8 or angiogenic factors including VEGF, Angiopoietin 36, 37. Additionally, exosome-mediated cell signaling might also play a role in the cardioprotection. However, the exact factors involved in these mechanisms remain unknown.

### Study limitations

The study had some noticeable limitations. Firstly, it was not randomized study and there were considerable differences in baseline parameters of patients in study groups which significantly impeded analysis of obtained results. Secondly, in our study, patients with STEMI were not homogenous groups with the same coronary artery obstructed. Thirdly, there was a limited number of recruited patients in both groups. Eventually, relatively low and variable number of LIN^-^ cells were obtained and then administered intracoronary in each patient.

## Conclusions

In conclusion, stem cell-based therapy for cardiac regeneration still needs more accurate and extensive investigations to estimate and improve their feasibility and efficacy. The obtaining and intracoronary infusion of autologous bone marrow-derived LIN^-^ stem/progenitor cells is feasible and safe for patient who experienced STEMI. There were no deaths, severe adverse cardiac effects, ventricular or supraventricular arrhythmias connected with procedure. 60% of patients from the LIN^-^ group (Responders) achieved an improvement in LV ejection fraction (>10% after 12 months) with no signs of unfavorable remodeling of LV from day 3^rd^ to 12^th^ month. Further studies are needed to assess long-term effects, potential side effects and to determine whether cell therapy has the potential to increase lifespan and decrease mortality.

## Figures and Tables

**Table 1:** The phenotypic characterization of administered autologous LIN^-^ cells.

	Phenotypic characterization	Function
**LIN^-^ cells**	CD2^-^, CD3^-^, CD11b^-^, CD14^-^, CD15^-^, CD16^-^, CD19^-^, CD56^-^, CD123^-^, CD235a^-^ (Glycophorin A)	The fraction does not contain any morphotic elements exhibiting mature phenotype. The depletion of lineage positive cells results in the enrichment of precursor cells, progenitor cells and stem cells such as CD34^+^ and CD133^+^ cells.

**Table 2:** Clinical characteristics of patients.

	LIN^-^ Group (n=15)	Standard therapy group (n=19)	p value
Age [y] mean (SD)	52.4 (7.2)	51.6 (8.5)	0.78
**Cardiovascular Risk Factors**			
Hypertension n (%)	5 (33.3)	6 (31.6)	0.91
Current smoking n (%)	8 (53.3)	10 (52.6)	0.97
Diabetes mellitus n (%)	0 (0.0)	0 (0.0)	-
Family history of CAD, n (%)	7 (46.7)	10 (52.6)	0.73
Body mass index, kg/m^2^	28.5	30.7	0.096
**Quantitative parameters**	**Mean (SD)**	**Mean (SD)**	**p value**
CKMB [U/l]	274.1 (186.9)	402.8 (245.4)	0.15
Maximum CKMB concentration [U/l]	709	927	
TN-I [µg/l]	15.2 (9.3)	18.9 (7.8)	0.23
Maximum TN-I concentration [µg/l]	30	29.6	
BNP [pg/ml]	1745.5 (1216.0)	1758.7 (1002.4)	0.78
Maximum BNP concentration [pg/ml]	6182	5088	
**Lipid profile**	**Mean (SD)**	**Mean (SD)**	**p value**
Total cholesterol [mg/dl]	238.0 (35.0)	212.0 (44.0)	0.10
LDL cholesterol [mg/dl]	149.0 (31.0)	143.0 (37.0)	0.70
HDL cholesterol [mg/dl]	49.0 (11.9)	43.9 (13.2)	0.17
Triglycerides [mg/dl]	212.0* (126.0)	139.0 (84.0)	0.02
**Ultrasonography**	**Mean (SD)**	**Mean (SD)**	**p value**
LVESV [ml]	83.1* (20.3)	98.3 (30.4)	0.04
EF [%]	38.5* (4.9)	35.3 (5.0)	0.05
LVEDV [ml]	134.3* (28.8)	156.9 (39.2)	0.03
TIMI flow grade after PCI	**n (%)**	**n (%)**	
3, n (%)	100	100	
**Qualitative parameters**	**n (%)**	**n (%)**	**p value**
Infarction site:			
Anterior	10 (66.7)	14 (73.7)	0.20
Inferior	4 (26.7)	4 (21.1)	
Lateral	1 (6.7)	1 (5.3)	
Supplied coronary artery:			
LAD n (%)	10 (66.7)	14 (73.7)	0.71
RCA n (%)	3 (20.0)	4 (21.1)	
LCX n (%)	2 (13.3)	1 (5.3)	

Mann-Whitney U test for quantitative variables or Fisher exact test for qualitative ones; p value - LIN^-^ group vs standard therapy group.

**Table 3:** Clinical characteristics of the study group divided into Responders and Non-responders.

	Responders group (n=9)	Non-responders group (n=6)	p value
Age [y] mean (SD)	52.8 (6.5)	51.8 (8.8)	0.95
**Cardiovascular Risk Factors**			
Hypertension n (%)	3 (33.3)	2 (33.3)	1.0
Current smoking n (%)	5 (55.5)	3 (50.0)	1.0
Diabetes mellitus n (%)	0 (0.0)	0 (0.0)	-
Family history of CAD, n (%)	4 (44.4)	3 (50.0)	1.0
Body mass index, kg/m^2^, mean (SD)	31.0 (3.7)	30.2 (2.9)	0.46
**Quantitative parameters**	**Mean (SD)**	**Mean (SD)**	**p value**
CKMB [U/l]	208.2* (198.2)	373.0 (123.8)	0.03
Maximum CKMB concentration [U/l]	709	527	
TN-I [µg/l]	10.4* (6.6)	22.4 (8.3)	0.01
Maximum TN-I concentration [µg/l]	20.3	29.6	
BNP [pg/ml]	1061.2* (484.5)	2772.0 (1287.3)	0.01
Maximum BNP concentration [pg/ml]	2193	4631	
**Lipid profile**	**Mean (SD)**	**Mean (SD)**	**p value**
Total cholesterol [mg/dl]	230.0 (35.2)	250.7 (33.6)	0.22
LDL cholesterol [mg/dl]	139.3 (28.4)	164.5 (30.4)	0.11
HDL cholesterol [mg/dl]	45.9 (12.6)	53.7 (9.8)	0.18
Triglycerides [mg/dl]	236.1 (155.8)	174.5 (55.3)	0.95
**Ultrasonography**	**Mean (SD)**	**Mean (SD)**	**p value**
LVESV [ml]	76.2 (15.8)	93.5 (23.3)	0.11
EF [%]	39.9 (4.9)	36.5 (4.4)	0.14
LVEDV [ml]	126.4 (22.7)	146.0 (34.8)	0.46
TIMI flow grade after PCI	**n (%)**	**n (%)**	
3, n (%)	100	100	
**Qualitative parameters**	**n (%)**	**n (%)**	**p value**
Infarction site:			
Anterior	6 (66.7)	5 (83.3)	
Inferior	3 (33.3)	0 (0.0)	
Lateral	0 (0.0)	1 (16.7)	
Supplied coronary artery:			
LAD n (%)	5 (55.5)	5 (83.3)	0.29
RCA n (%)	3 (33.3)	0 (0.0)	
LCX n (%)	1 (11.1)	1 (16.7)	

Mann-Whitney U test for quantitative variables or Fisher exact test for qualitative ones; p value - Responders group vs Non-responders group.

**Table 4:** The selected ultrasonographic parameters in Responders group and in Non-responders group (0, 1^st^, 3^rd^, 7^th^ day, and in the 1^st^, 3^rd^, 6^th^, 12^th^ month).

	0 day	1^st^ day	3^rd^ day	7^th^ day	1^st^ month	3^rd^ month	6^th^ month	12^th^ month
**EF [%]**								
Responders group	40.0 (5,8)	41.5 (6.0)	45.5 (8.5)	49.0* (5.0)	50.0* (6.8)	51.1* (4.3)	55.0* (2.8)	55.0* (2.5)
Non-responders group	35.0 (8.0)	37.0 (6.5)	38.0 (8.5)	40.0* (8.0)	37.0* (11.0)	37.0* (8.5)	37.0* (9.5)	37.0* (6.5)
p value	0.14	0.18	0.018	0.008	0.008	0.018	0.001	0.18
**LVEDV [ml]**								
Responders group	128.5 (34.8)	130.5 (45.5)	130.5 (44.5)	122.5 (34.8)	116.5 (17.0)	110.5 (17.3)	100.0 (21.8)	101.5 (29.0)
Non-responders group	125.0 (53.0)	120.0 (39.0)	120.0 (34.5)	120.0 (33.5)	129.0 (31.0)	142.0 (42.5)	150.0 (37.5)	136.0 (45.0)
p value	0.46	0.95	0.95	0.95	0.27	0.049	0.002	0.003
**LVESV [ml]**								
Responders group	72.5 (16.3)	71.5 (8.0)	68.5 (9.5)	60.5 (12.0)	57.5 (7.8)	48.0 (14.3)	42.5* (12.0)	42.0 (15.3)
Non-responders group	76.0 (35.5)	72.0 (24.0)	76.0 (33.0)	72.0 (30.0)	74.0 (26.5)	83.0 (35.0)	82.0* (24.5)	84.0 (36.0)
p value	0.011	0.46	0.27	0.22	0.11	0.012	0.003	0.002
**LVIDD [mm]**								
Responders group	51.0 (3.3)	51.5 (9.0)	49.5* (8.3)	49.5* (8.0)	49.5 (7.8)	50.0 (8.5)	47.5* (2.0)	48.5 (11.3)
Non-responders group	53.0 (7.5)	50.0 (6.5)	56.0* (3.5)	57.0* (3.0)	55.0 (2.0)	58.0 (9.5)	58.0* (5.0)	57.0 (6.0)
p value	0.39	1.0	0.026	0.001	0.39	0.018	0.001	0.066

Data are expressed as median (IQR); p value - Responders group vs Non-responders group, Mann-Whitney U-test; * p < 0.05 for difference between 0 day and subsequent time points, Friedman ANOVA followed by Wilcoxon signed-rank test; for all differences significant in Wilcoxon signed-rank test, Friedman ANOVA also yielded p < 0.05.

**Table 5:** Troponin I, CK-MB and BNP levels in Responders group and in Non-responders group (0, 1^st^, 3^rd^, 7^th^, and in the 1^st^, 3^rd^, 6^th^, 12^th^ month).

	0 day	1^st^ day	3^rd^ day	7^th^ day	1^st^ month	3^rd^ month	6^th^ month	12^th^ month
**Troponin I [µg/l]**								
Responders group	7.3 (4.3)	2.5 (1.7)	1.0 (0.4)	0.2 (0.2)	0.01 (0.0)	0.01 (0.0)	0.01 (0.0)	0.01 (0.0)
Non-responders group	24.4 (14.2)	8.3 (4.7)	4.1 (1.8)	0.6 (0.6)	0.01 (0.0)	0.01 (0.0)	0.01 (0.0)	0.01 (0.0)
p value	0.012	0.008	0.008	0.004	1.0	1.0	1.0	1.0
**CK-MB [IU/l]**								
Responders group	136 (65)	35 (16)	24 (9)	18 (6)	17 (3)	15 (4)	15 (3)	18 (4)
Non-responders group	361 (190)	71 (56)	31 (20)	17 (10)	16 (4)	16 (5)	16 (4)	17 (4)
p value	0.026	0.012	0.33	0.61	0.86	0.61	0.22	0.53
**BNP [pg/ml]**								
Responders group	834 (503)	909 (418)	632 (258)	370 (280)	542 (594)	315 (262)	104 (83)	87 (61)
Non-responders group	2298 (1919)	1370 (1476)	1387 (1285)	1034 (1141)	1435 (857)	805 (413)	468 (389)	361 (350)
p value	0.008	0.088	0.002	0.001	0.005	0.012	0.012	0.008

Data are expressed as median (IQR); p value - Responders group vs Non-responders group.

## Abbreviations

LV: left ventricle
SPCs: stem/progenitor cells
STEMI: ST-elevated myocardial infarct
LIN^-^: lineage-negative
BM: bone marrow
BNP: brain natriuretic peptide
IRA: infarct-related artery
AMI: acute myocardial infarction
CK-MB: creatine kinase
LVEF: left ventricular ejection fraction
PCI: percutaneous coronary intervention
ECG: electrocardiogram
EF: ejection fraction
DES: drug eluting stent
MNCs: mononuclear cells
PBS: phosphate buffered saline
MACE: major adverse cardiac events
LVEDV: end-diastolic volume
LVESV: end-systolic volume
IQR: interquartile rang
LAD: anterior descending artery
LCX: left circumflex artery
RCA: right coronary artery
LVIDD: left ventricular internal dimension at end-diastole
SD: standard deviation
VEGF: vascular endothelial growth factor
SDF-1: stromal derived factor-1
MSCs: mesenchymal stem cells
IL: interleukin
